# Supplementary figures and images for: Lung Epithelial Signaling Mediates Early Vaccine-Induced CD4+ T Cell Activation and Mycobacterium tuberculosis Control
Source: mBio. 2021 Jul 13;12(4):e01468-21. doi: 10.1128/mBio.01468-21 (PMC8406195; doi:10.1128/mBio.01468-21)

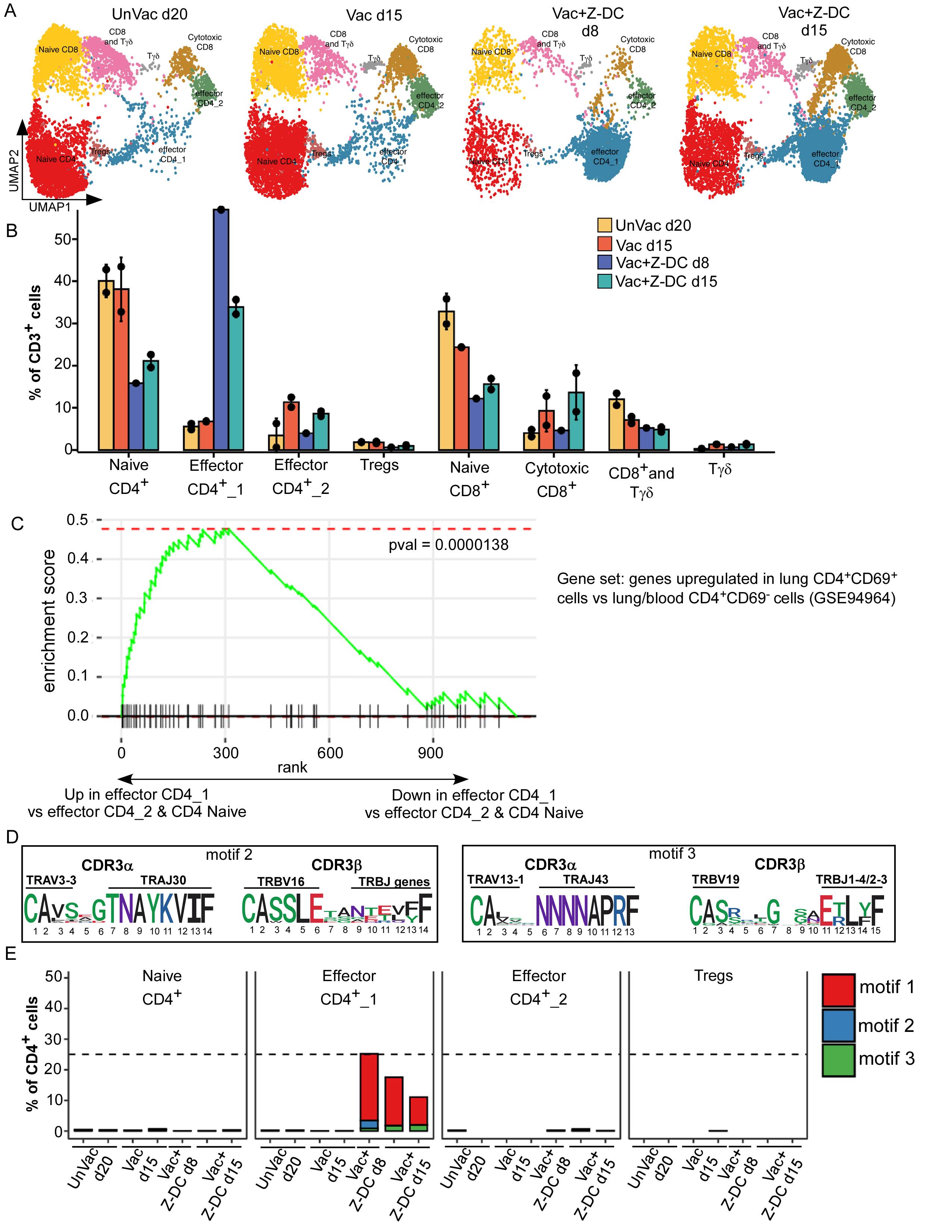

Supplement: FIG S1 [file mbio.01468-21-sf001.tif]

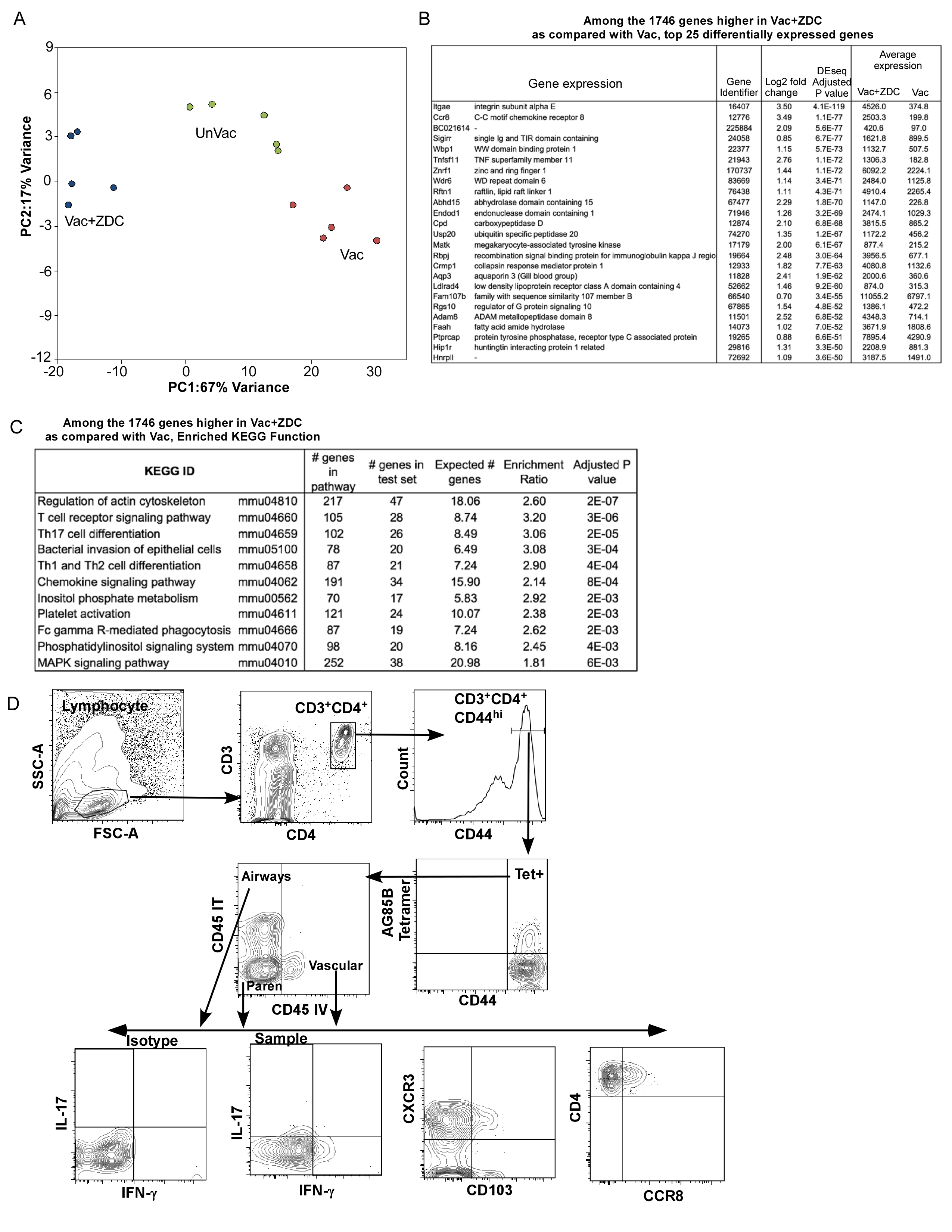

Supplement: FIG S2 [file mbio.01468-21-sf002.tif]

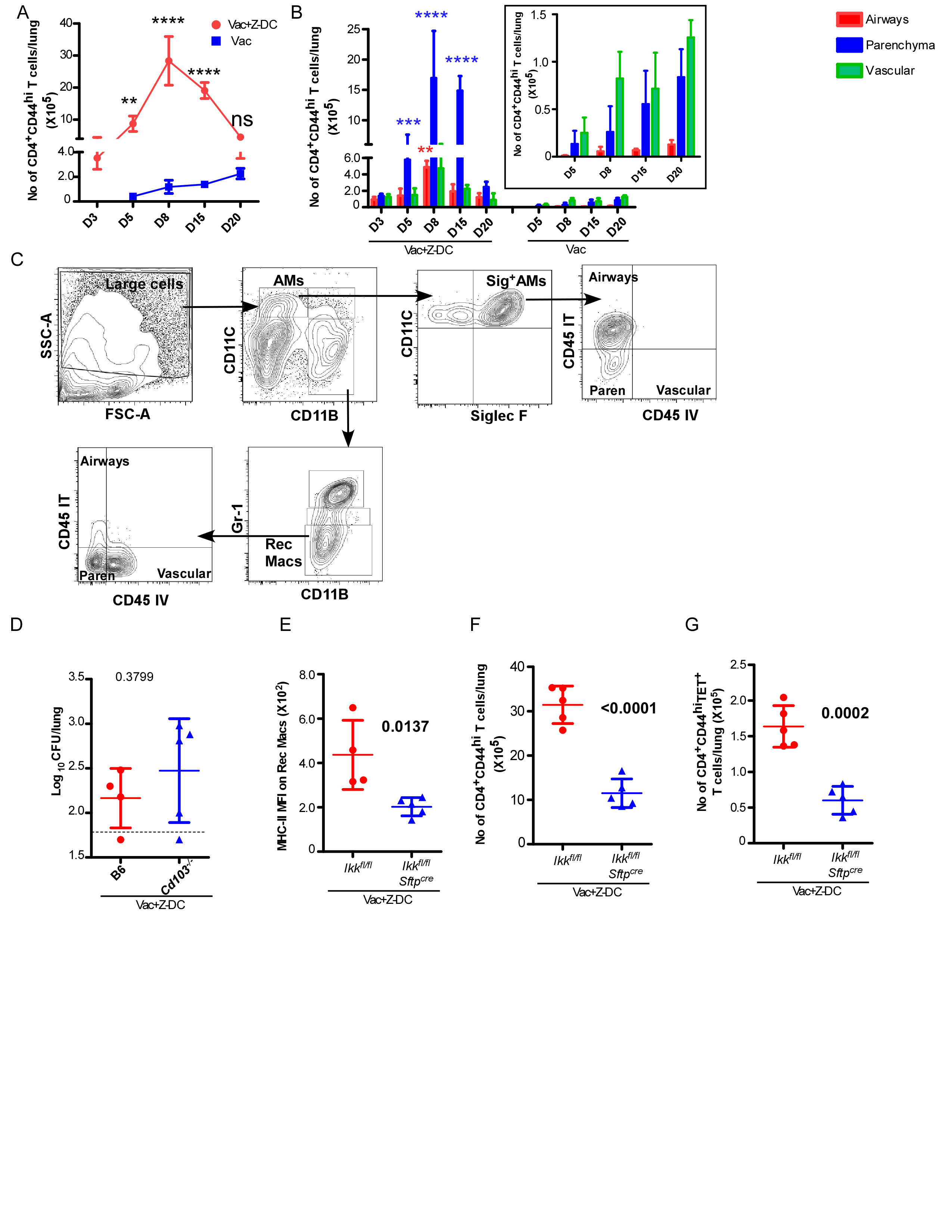

Supplement: FIG S3 [file mbio.01468-21-sf003.tif]
